# Comparing the Lifestyle Interventions for Prediabetes: An Integrated Microsimulation and Population Simulation Model

**DOI:** 10.1038/s41598-019-48312-z

**Published:** 2019-08-15

**Authors:** Amin Khademi, Lu Shi, Amir Ali Nasrollahzadeh, Hariharaprabhu Narayanan, Liwei Chen

**Affiliations:** 1 Department of Industrial Engineering, Clemson University, Clemson, 29634 USA; 2 Department of Public Health Sciences, Clemson University, Clemson, 29634 USA; 3 Department of Epidemiology, UCLA Fielding School of Public Health, Los Angeles, 90095 USA

**Keywords:** Health policy, Risk factors

## Abstract

We developed a model to compare the impacts of different lifestyle interventions among prediabetes individuals and to identify the optimal age groups for such interventions. A stochastic simulation was developed to replicate the prediabetes and diabetes trends (1997–2010) in the U.S. adult population. We then simulated the population-wide impacts of three lifestyle diabetes prevention programs, i.e., the Diabetes Prevention Program (DPP), DPP-YMCA, and the Healthy Living Partnerships to Prevent Diabetes (HELP-PD), over a course of 10, 15 and 30 years. Our model replicated the temporal trends of diabetes in the U.S. adult population. Compared to no intervention, the diabetes incidence declined 0.3 per 1,000 by DPP, 0.2 by DPP-YMCA, and 0.4 by HELP-PD over the 15-year period. Our simulations identified HELP-PD as the most cost-effective intervention, which achieved the highest 10-year savings of $38 billion for those aged 25–65, assuming all eligible individuals participate in the intervention and considering intervention achievement rates. Our model simulates the diabetes trends in the U.S. population based on individual-level longitudinal data. However, it may be used to identify the optimal intervention for different subgroups in defined populations.

## Introduction

Over 25 million Americans have diabetes, with approximately 2 million new diagnoses annually^1^. Complications associated with diabetes incur significant costs to individuals, families, and health care systems^2^. The costs of diabetes in the U.S. was $255 billion in 2012, a 41% increase since 2007^3^. Prediabetes represents a stage when the blood glucose exceeds the optimal level but has not met the diagnosis criteria of diabetes^4^. Emerging evidence from randomized controlled clinical trials demonstrated that lifestyle interventions for prediabetes individuals can delay the onset of diabetes^5–9^, and reduce total and cardiovascular mortality^10^. Thus, individuals with prediabetes represent a unique high-risk population that might benefit from lifestyle interventions without pharmacological treatment. In 2017, the Centers for Disease Control and Prevention (CDC) reported that 84.1 million U.S. adults are estimated to have prediabetes, which often progresses to diabetes within a few years if untreated^11^. However, interventions for prediabetes require substantial resources and it is not yet clear how to allocate public health and clinical resources to minimize diabetes incidence. Questions remain on whether the benefits of such interventions sustain beyond the active treatment period and whether such interventions can reduce mortality rate. Seeking cost-effective interventions targeted on prediabetes individuals that can be conducted in clinical or community settings is a top priority for fighting the diabetes epidemic.

Computer models simulating the impacts of screening, lifestyle interventions, or different treatments are used to assess the effectiveness, costs, complications, and quality of life improvements. Several studies used Markov/semi-Markov chains to model the progression of diabetes status in an individual or population^12–15^. These studies used transition probabilities to model the change in the disease progression, whereas our framework considers a microsimulation model to incorporate heterogeneity, compare efficacy and cost-effectiveness of a variety of lifestyle interventions among the U.S. adult population, as well as identify optimal age groups for each intervention. Our model is different from other diabetes models in the following ways. First, the transition probabilities between disease stages were built upon individual-level data rather than a parameter value extracted from literature. This feature enables us to model the progression of each individual reflecting unique demographic characteristics. Second, our model simulates the entire life history of each individual, which allows us to explore the optimal age range of different interventions. Third, our life course approach estimates the gain in diabetes-free survival time for any given intervention, i.e., the average time for an individual with prediabetes conditions to develop diabetes.

## Methods

**Figure 1:**
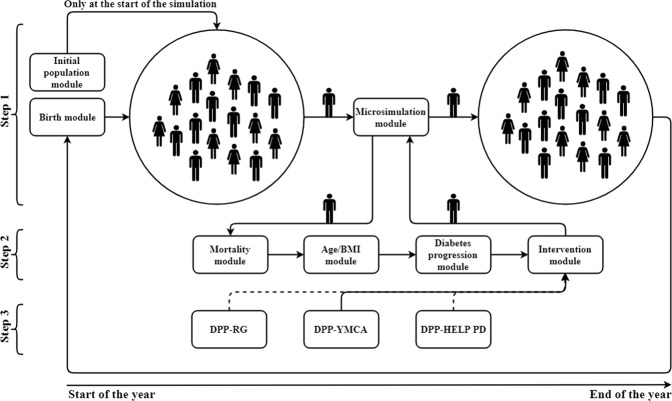
Overview of the simulation model.

We developed a stochastic population-based simulation model to replicate prediabetes and diabetes trends in the U.S. adult population from 1997 to 2010. The stochastic progression of each individual is based on a microsimulation model for which the transition probability matrix (TPM) was estimated with data obtained from the PREMIER study^16^. The microsimulation and population simulation models were validated in several dimensions. Most importantly, the population simulation model was validated for the U.S. adult population from 1997 to 2010 (see Supplement A). To estimate and validate the efficacy of multiple intervention strategies, we extended the model for a course of 5 (2010 to 2015), 10 (2010 to 2020), and 15 (2010 to 2025) years and simulated the diabetes trends for three lifestyle interventions^5,17,18^. The efficacy was evaluated by comparing the population progression outcomes in intervention groups with those of a control group for categories such as diabetes incidence, prevalence, and mortality rates. To investigate cost-efficiency, the simulation is extended for a course of 10 (2010 to 2020) and 30 (2010 to 2040) years to evaluate averted cases of death and diabetes, as well as the average delay for diabetes onset (i.e., gain in diabetes-free survival time). We described the population-based simulation and the microsimulation in the following steps. Figure 1 demonstrates an overview of both simulation models, their modules, and the relationship between the population-based simulation model and the microsimulation model.

### Population-based simulation

We developed a population-based model to estimate and compare the impacts (both health and economic outcomes) of lifestyle interventions on prediabetes and diabetes trends. We used object-oriented programming to develop the population-based simulation, where progression of each individual is governed by the microsimulation. The population-based simulation model consists of an Initial Population Module to generate initial population, a Birth Module which simulates the population growth, and a microsimulation model, described in Step 2 (Fig. 1), to simulate the progression of each individual.

#### Initial population module

We simulated U.S. adult (>20) population in 1997 based on distributions of age, gender, body mass index (BMI), and diabetes status^19–21^. We validated this module by comparing the simulated population for each demographic characteristic with the corresponding U.S. population between 1997 and 2010 (Supplementary Table S1).

#### Birth module

Each year, “birth” to the population is computed as the number of those turning 20 years old, calculated from available data sets (Supplementary Table S8). The distribution of newly added individuals in each year (with respect to age, gender, BMI, and diabetes status) corresponds to that of the U.S. population at that year. We validated the population-based simulation model from 1997 to 2010 in five dimensions: total population (Supplementary Table S1), diabetes incidence (Supplementary Table S2), diabetes prevalence (Supplementary Table S3), prediabetes prevalence (Supplementary Table S4), and mortality rate (Supplementary Table S5). Therefore, the overall simulation model describes the complex progression of individuals from prediabetes to diabetes correlating the progression to the distributions of BMI in each age-gender category.

### Microsimulation

Microsimulation is characterized by specifying the probabilistic progression of each individual based on demographic characteristics. Each individual is characterized by age, gender, BMI, and fasting glucose level (FGL). We discretized age to 17 groups: 20–25, 25–30, .…., 100+; BMI to three groups: “Normal weight” (BMI between 20–25 kg/m^2^), “Overweight” (BMI between 25–30 kg/m^2^), and “Obese” (BMI greater than 30 kg/m^2^); FGL to three groups: “No diabetes” (less than 100 mg/dl), “Prediabetes” (between 100–125 mg/dl), and “Diabetes” (greater than 125 mg/dl). The microsimulation model consists of a Mortality Module, which decides on termination of life for each individual, an Intervention Module described in details in Step 3 (Fig. 1), which simulates the interventions’ effects, an Age/BMI Module, which increments age and replicates BMI trends, and a Diabetes Progression Module in which progression between diabetes statuses “No Diabetes,” “Prediabetes,” and “Diabetes” is simulated. To calibrate and/or validate each module, the microsimulation model is run from 1997 to 2010, where for each individual at baseline (1997), age and gender were assigned based on a cumulative density function (CDF) obtained from the 1997 census^22^. BMI and FGL are assigned based on a CDF obtained from CDC data described in details below^23,24^.

#### Mortality module

At the beginning of each year, the model determines whether an individual will be alive or dead at the end of that year. To that end, for individuals whose diabetes status is “No diabetes” or “Prediabetes”, a random number between zero and one is generated and calibrated to the CDF of age-gender-specific U.S. adult mortality data^25^. This module is validated by comparing life expectancies generated by the mortality module and CDC estimations in the same period (Supplementary Table S6). For diabetes patients, a random number between zero and one is generated and calibrated to the mortality rate produced by a validated Cox proportional-hazards model for diabetic individuals^26^. Note that the original Cox model contained covariates such as cancer history which are not considered in our model. Therefore, the model is adjusted by adding a constant (i.e., changing the baseline hazard) to compensate for the effects of missed factors and is calibrated with respect to the U.S. population data^27^ (Supplementary Table S7).

#### Age/BMI module

At each time period, age is incremented deterministically, i.e., age at period (*t* + 1) is age at period (*t*) + 1. BMI values change stochastically based on a regression model constructed from the reported data of the average BMI trend for each gender in the U.S.^19–21^. The module also decided whether to change the BMI of an individual in each period. Consequently, the amount of change is determined based on a probability distribution whose expected value equals the rate of BMI change produced by the regression model (Supplementary Table S9).

#### Diabetes progression module

The FGL progression in an individual is modeled by a Markov chain with three states: (1) “No diabetes” (less than 100 mg/dl), (2) “Prediabetes” (between 100 and 125 mg/dl), and (3) “Diabetes” (greater than 125 mg/dl). Markov chains are a common modeling technique to describe the probabilistic progression of patients’ health over time^28^. We constructed TPMs using the longitudinal data from the PREMIER study (see Supplement B).

### Intervention module

Although the model is flexible to simulate a variety of interventions, we simulated the impacts of three proven lifestyle interventions DPP, DPP-YMCA, and HELP-DP (the latter two being the translational versions of DPP) for 10 and 30 years starting from 2010. These interventions were chosen for simulation here because they are U.S.-based diabetes prevention interventions with proven effectiveness and publicly available cost information^29–32^. In simulating these interventions, we did not reduce BMI for individuals whose BMI was below 18.5 (underweight). In the first year of the microsimulation model, an intervention is simulated by reducing the BMI of eligible individuals. This reduction is only sustained for one year. After the first year, each individual’s BMI progresses according to an individual-level stochastic model. To have a fair comparison, we considered the same sub-population for all interventions: age 25 to 65, BMI 25+, and an elevated FGL. We also conducted a separate simulation study where each intervention is simulated for the population that is associated with its inclusion criteria (see Supplement C).

#### Diabetes prevention program (DPP)

DPP is a randomized control trial where prediabetes participants are assigned to three groups: placebo, metformin treatment, or lifestyle intervention^5^. We only simulated the lifestyle intervention which achieved at least 7% BMI reduction for 38% of participants measured some time after the first year of the trial. One may interpret the 38% figure as the achievement rate for the intervention, which is considered for other interventions as well.

#### DPP-young men’s christian association (DPP-YMCA)

DPP-YMCA adapted the DPP to group sessions and delivered them through Young Men’s Christian Association^17^. We simulated the trial’s lifestyle intervention which achieved at least 5% BMI reduction for 32.4% of participants after one year.

#### Healthy-living partnerships to prevent diabetes (HELP-PD)

HELP-PD is a diabetes education program delivered by community health workers^18,33^. We simulated the diabetes education intervention, which achieved at least 5% BMI reduction for 58.5% of participants after one year.

## Analysis

We considered three performance measures: (1) Diabetes Incidence, which is the number of new cases of diabetes in each year divided by the population, excluding those who had been previously diagnosed with diabetes; (2) Diabetes Mortality, which is the number of diabetes death cases divided by the population; and (3) Diabetes Prevalence, which is the total number of diabetes cases divided by the population. To estimate and validate efficacy, we simulated the impacts of DPP, DPP-YMCA, and HELP-PD interventions over a course of 5, 10, 15 years in the U.S. adult population starting from 2010 using the same target population for each intervention. The improvement percentage for a particular performance measure is calculated according to $\frac{({{\rm{rate}}}_{I,15}-{{\rm{rate}}}_{NI,15})}{{{\rm{rate}}}_{NI,15}}$, where rate_*I*,15_ and rate_*NI*,15_ denote the performance measure of the intervention and no-intervention groups at the end of 15 years, respectively. To compare cost-effectiveness and test the robustness of the optimal target population over time, we simulated 10-year (short-term) and 30-year (long-term) performance of each intervention in terms of total number of death cases averted, total number of diabetes cases averted, and average gain in diabetes-free survival time in four different age groups (25–65, 35–65, 45–65, and 55–65 years). The aversion in death and diabetes cases refer to the difference in number of death and new diabetes cases in the intervention and control population. We conducted an analysis to identify the optimal age group that produces minimal diabetes cases with maximum cost savings. The optimal target population was defined as the one that achieves maximum cost savings, calculated by the number of diabetes averted multiplied by the benefit of averting diabetes (medical cost associated with diabetes) minus the total cost of implementing the intervention. The cost for each intervention per individual in DPP, DPP-YMCA, and HELP-PD is obtained from literature^29–31^, as are the age-gender weighted average lifetime medical costs associated with diabetes^13^. We estimated the gain in diabetes-free survival time attributable to lifestyle interventions. We created two identical sets of 2010 U.S. populations in the population-based simulation model and applied each intervention to one of the populations and treated the other population as a control. We then kept track of the times required for an individual to develop diabetes in both populations. In the case an individual developed diabetes in both populations, the time when the individual developed diabetes in the intervention group was subtracted from that of the control group. If an individual developed diabetes in neither of the populations, then the delay is set to zero. In the case an individual developed diabetes in one population and not in the other, if the individual developed diabetes in the control population, the final simulation time was subtracted from the diabetes development time and the difference was reported as an estimate for the gain in diabetes-free survival time. However, if the individual developed diabetes in the intervention population, the time of diabetes development is subtracted from the final simulation time and is reported as an estimate for the loss in diabetes-free survival time. Finally, if an individual dies before developing diabetes in one population, then we exclude the individual from analysis.

Our simulation models are coded using C++ which are available by request. Parameter inputs and calculations are provided in Supplement B, and Supplementary Tables S9–S11.

## Results

**Figure 2:**
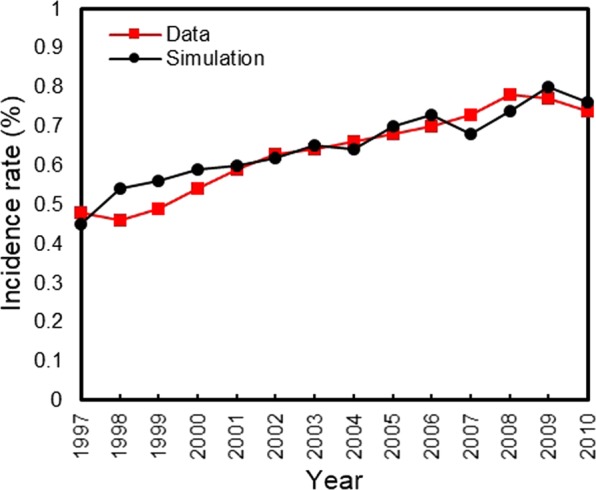
Comparing U.S. diabetes incidence rate from 1997 to 2010.

**Figure 3:**
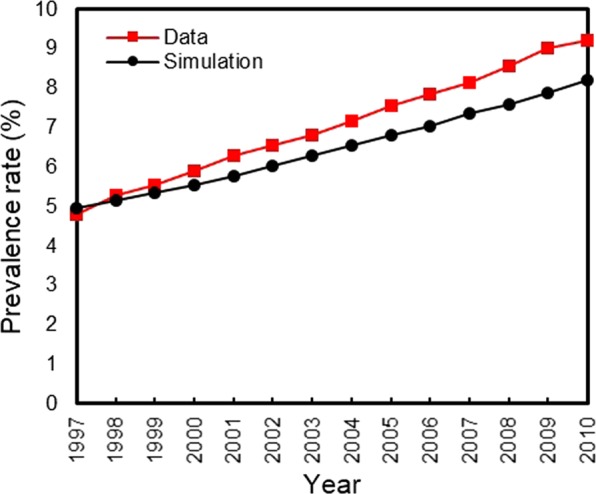
Comparing U.S. diabetes prevalence rate from 1997 to 2010.

Our simulated population of U.S. adults (>20 years) corresponded well to the observed population from 1997 to 2010 (see Supplement A). Our Initial Population Module is validated by comparing total U.S. adult population (Supplementary Table S1) and total U.S. population by different age groups, BMI and diabetes status (Supplementary Figs S1–S3). The model slightly underestimated the diabetes prevalence rate (Supplement A), which is likely to make conservative contributions to intervention performance. The Birth Module is validated by diabetes incidence, diabetes prevalence, prediabetes prevalence, and death cases over time^34^ (Figs 2, 3, and Supplementary Tables S2–S5). Finally, our Mortality Module is validated by comparing life expectancies with respect to diabetes status (Supplementary Tables S6 and S7).

### Performance measures

Three performance measures (differences in diabetes incidence, diabetes prevalence, and mortality) after applying three interventions for one year as compared with a simulated control population (without intervention) in a course of 5, 10, and 15 years are presented in Table 1. The reduction in diabetes incidence (without intervention - with intervention) over 5, 10, and 15 years was 0.06, 0.03, and 0.03 per 100 by DPP, 0.03, 0.02, and 0.02 per 100 by DPP-YMCA, and 0.06, 0.04, and 0.04 per 100 by HELP-PD. Such reductions are about an improvement in diabetes incidence over 5, 10, and 15 years of 6.25%, 2.96%, and 2.99% by DPP, 3.39%, 1.91%, and 2.16% by DPP-YMCA and 6.79%, 3.38%, and 3.28% by HELP-PD. The reduction in diabetes prevalence over 5, 10, and 15 years were 0.18, 0.28, and 0.33 per 100 by DPP, 0.12, 0.2, and 0.2 per 100 by DPP-YMCA, and 0.2, 0.31, and 0.35 per 100 by HELP-PD. The reduction in mortality rates due to diabetes after 5, 10, and 15 years were 0.01, 0.02, and 0.03 per 100 in DPP, 0.0, 0.01, and 0.01 per 100 in DPP-YMCA, and 0.01, 0.02 and 0.03 per 100 by HELP-PD.

**Table 1:** Differences and improvement percentages in diabetes incidence, prevalence and mortality rate of lifestyle interventions compared to no intervention in the U.S. adult population after 5, 10, and 15 years.

	Intervention Type	Differences (%)			Improvements (%)		
		5 yrs	10 yrs	15 yrs	5 yrs	10 yrs	15 yrs
Diabetes incidence	DPP	0.06	0.03	0.03	6.25	2.96	2.99
	DPP-YMCA	0.03	0.02	0.02	3.39	1.91	2.16
	HELP-PD	0.6	0.04	0.04	6.79	3.38	3.28
Diabetes prevalence	DPP	0.18	0.28	0.33	1.55	2.06	2.2
	DPP-YMCA	0.12	0.2	0.2	1	1.32	1.32
	HELP-PD	0.2	0.31	0.35	1.68	2.27	2.44
Diabetes mortality	DPP	0.01	0.02	0.03	1.41	2.29	4.65
	DPP-YMCA	0.00	0.01	0.01	0.53	0.98	2.92
	HELP-PD	0.01	0.02	0.03	1.62	3.39	5.23

Note: Difference (%) = % without intervention − % with intervention.

Improvement (%) = (% without intervention − % with intervention)/% without intervention.

### Intervention impacts

In the short-term (10-year) and long-term (30-year) simulations, diabetes costs are evaluated by tracking the number of interventions. Savings are evaluated by enumerating diabetes cases averted. Table 2 shows short-term impacts of each intervention by age group (25–65, 35–65, 45–65, and 55–65). Sustaining the BMI reduction resulted by DPP intervention for only one year among prediabetes patients aged between 55–65 years averted 258,700 diabetes incidents and gained, on average, 0.03 years of diabetes-free survival. It averted 182,400 deaths in this population but it resulted in approximately $9 billion of costs over 10 years. Moreover, if DPP was offered to younger populations (e.g. 45–55, 35–65 or 25–65 years), more substantial impacts would have been gained (Table 2) while costing substantially more as well. However, the HELP-PD intervention appears to have nearly the same impact in every age group. This is important because the HELP-PD intervention will eventually results in substantial savings in contrast to the DPP intervention. Table 2 shows that the optimal configuration is a combination of the HELP-PD intervention for 25–65 years individuals. The 30-year impacts of all interventions (Supplement C) also confirm these results. However, these results, particularly in the long-term, should be interpreted with consideration of modeling assumptions, which will be discussed in depth in Discussion Section.

**Table 2:** Short-term (10 years) cost-effectiveness analysis for lifestyle interventions among individuals with prediabetes.

	Intervention Type		
	DPP	DPP-YMCA	HELP-PD
Age group		55–65 yrs	
Diabetes averted (1000 s)	258.78	150.35	226.00
Death averted (1000 s)	18.24	4.96	22.80
Gain in diabetes-free survival time (Years)	0.03	0.02	0.03
Intervention cost ($1000)	31,074,700	7,677,100	10,039,300
Aversion savings ($1000)	22,048,056	12,809,820	19,255,200
Total savings ($1000)	−9,026,644	5,132,720	9,215,900
Age group		45–65 yrs	
Diabetes averted (1000 s)	489.06	296.67	509.60
Death averted (1000 s)	44.08	26.35	58.00
Gain in diabetes-free survival time (Years)	0.06	0.03	0.06
Intervention cost ($1000)	62,174,800	15,361,400	20,088,000
Aversion savings ($1000)	41,667,912	25,276,284	43,417,920
Total savings ($1000)	−20,506,888	9,914,884	23,329,920
Age group		35–65 yrs	
Diabetes averted (1000 s)	652.84	394.32	764.00
Death averted (1000 s)	77.14	44.95	107.60
Gain in diabetes-free survival time (Years)	0.07	0.05	0.08
Intervention cost ($1000)	92,405,900	22,829,300	29,853,700
Aversion savings ($1000)	55,621,968	33,596,064	65,092,800
Total savings ($1000)	−36,783,932	10,766,764	35,239,100
Age group		25–65 yrs	
Diabetes averted (1000 s)	915.80	553.35	927.60
Death averted (1000 s)	83.98	62.31	130.40
Gain in diabetes-free survival time (Years)	0.11	0.06	0.11
Intervention cost ($1000)	126,929,900	31,358,600	41,007,400
Aversion savings ($1000)	78,026,160	47,145,420	79,031,520
Total savings ($1000)	−48,903,740	15,786,820	38,024,120

## Discussion

First of all, the results must be interpreted considering modeling assumptions and limitations. For example, in the Intervention Module, we simulated each intervention assuming achievement rates, which determines the proportions of individuals who adopt the intervention. This is because the individual adherence rate is not available and the full effect of adherence or intensity of participation is not known on BMI reduction in order to form a mathematical model. Therefore, we conservatively compensated for this effect by simulating the achievement rate where only a proportion of participants in the trial receive the full benefit and the rest do not. The effect of the intervention is immediately realized and is sustained uniformly for only a year. Moreover, when applying the intervention effects, all the participants are treated at the same time. We also assumed that all benefits of the interventions are realized with BMI reduction, which could underestimate their overall benefits. Lifestyle interventions may improve the diet quality or increase physical activity, which could reduce diabetes incidence without modifying BMI.

Recall that the progression of each individual in the population-based simulation is governed by a microsimulation model. However, this model which stochastically changes an individual’s BMI and FGL is calibrated with respect to a set of limited covariates: age, gender, BMI, and FGL. This is also the case for the Mortality Module which determines the probability of death for each individual. The model’s mortality risk for diabetes patients is based on a validated Cox proportional hazards model, which originally contains a broad set of risk factors. We eliminated several risk factors such as family history, physical inactivity, and race/ethnicity because the data sets were not large enough to accurately estimate them. However, their effect is estimated by adjusting the regression model. Moreover, the same techniques described in this study can be applied to construct transition probabilities and formulate stochastic changes in BMI value with respect to any combination of risk factors.

Therefore, when considering long-term results (Supplementary Table S12), the effects of these assumptions may be multiplied, and thus there is more uncertainty associated with long-term results with respect to short-term results. Also, we did not build in promotion campaign costs and facility investment costs associated with nationwide scale of interventions. This may be another contributing factor to uncertainty about the cost-effectiveness results because intervention costs or aversion savings are monetary values which are not accurately predictable in the long run.

We developed a parsimonious model to measure the potential impacts of applying a variety of preventive lifestyle interventions on diabetes incidence and prevalence rates, as well as diabetes mortality rate. The simulation model was validated by replicating observable trends of diabetes in the U.S. adult population. Our analysis showed that applying certain lifestyle interventions can significantly decrease the incidence and prevalence of diabetes, as well as mortality. Our results suggested that a lifestyle intervention, if sustained for one year, could significantly reduce diabetes prevalence over 15 years. The 15-year scenario showed that an intervention of any of the three types results in significant reductions in diabetes incidence, mortality, and prevalence. Moreover, long-term and short-term simulations indicated that the HELP-PD intervention achieves 5% BMI reduction for a higher percentage of participants and proved to be more efficacious in the 15-year scenario, is the most cost-effective lifestyle intervention. The optimal age group, in terms of cost-effectiveness, for such an intervention is 25–65 years.

Our model introduces several novel characteristics. First, its parameters are estimated with respect to individual-level data of a well-implemented clinical study with excellent follow-up rate (90%) and high-quality data collection and is calibrated to reflect the U.S. population trends of diabetes. Second, the microsimulation model simulates the individual life history of every person in the population and then reports the aggregate outcome, thus facilitating the identification of subgroups which are more likely to benefit from a health intervention in a given population. Such distributional patterns of health intervention benefits are key to future studies of minority health and health disparity. Third, our microsimulation model estimates the gain in diabetes-free survival time, as it simulates the life history of diabetes for each individual. Finally, our model can be easily applied to other defined inventions and other non-infectious diseases. Our calibration and validation have set up a sound baseline model for simulating population-level health interventions.

In conclusion, our study shows how a microsimulation-based model compares the cost-effectiveness of different diabetes prevention programs for decision makers weighing which intervention to adopt. Our model-based results, by incorporating attendance rate, corroborate with that of an earlier model that a proven weight loss program could save Medicare $7 billion or more^35^. Investing in the diabetes prevention programs among pre-retirement-age prediabetes patients might be motivated by both disease control and public finance.

## Supplementary information

Supplement file
