# Supplementary material for: Comparing the Lifestyle Interventions for Prediabetes: An Integrated Microsimulation and Population Simulation Model
Source: Sci Rep. 2019 Aug 15;9:11927. doi: 10.1038/s41598-019-48312-z (PMC6695408; doi:10.1038/s41598-019-48312-z)
Supplement: Supplementary file 1 — Supplement file [file 41598_2019_48312_MOESM1_ESM.pdf]

Comparing the Lifestyle Interventions for Prediabetes: An Integrated Microsimulation and Population Simulation Model - Supplementary Material

Amin Khademi[1,*] , Lu Shi[2], Amir Ali Nasrollahzadeh[1], Hariharaprabhu Narayanan[1], and Liwei Chen[3]

[1]Department of Industrial Engineering, Clemson University, Clemson, 29634, USA

[2]Department of Public Health Sciences, Clemson University, Clemson, 29634, USA

[3]Department of Epidemiology, UCLA Fielding School of Public Health, Los Angeles, 90095, USA

Supplement A. Validation Results

Validation of Initial Population Module

Supplement Table S1. Compare the simulated populations to U.S. adult populations between 1997 and 2010[1–3]

Year	Simulated adult population (1000s)	Real adult population (1000s)	Difference (%)
1997	194,154	194,110	0.02
1998	196,490	196,533	0.02
1999	198,846	199,000	0.08
2000	201,449	201,587	0.07
2001	203,857	204,062	0.10
2002	206,183	206,451	0.13
2003	208,306	208,682	0.18
2004	210,541	211,050	0.24
2005	212,975	213,511	0.25
2006	215,370	216,055	0.32
2007	217,693	218,481	0.36
2008	220,016	220,975	0.43
2009	222,426	223,491	0.48
2010	224,893	226,113	0.54

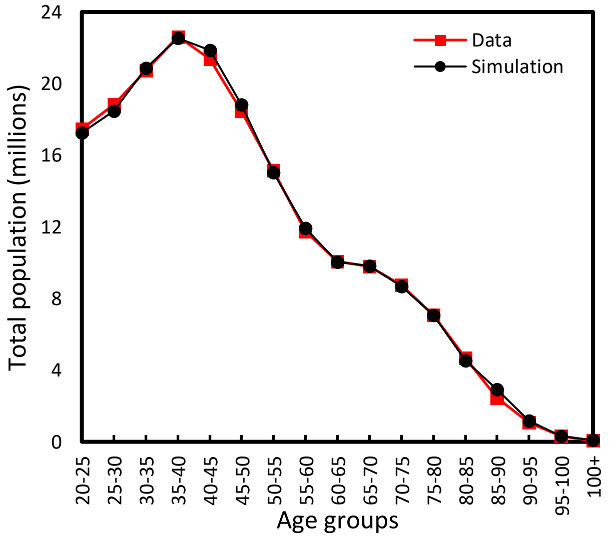

A

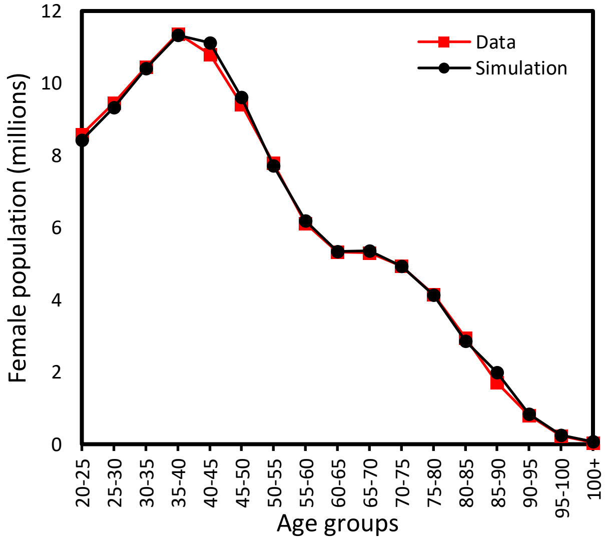

B

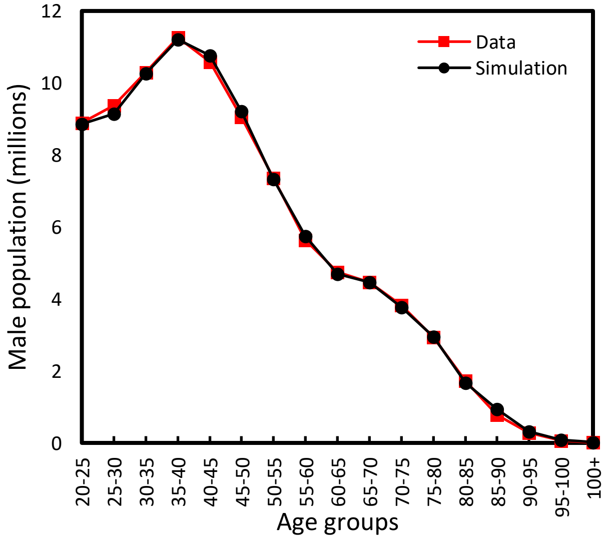

C

Supplement Figure S1. Comparing U.S. adult population in 1997 and the simulated population across age and by gender. A: Total population; B: Male population; C: Female population

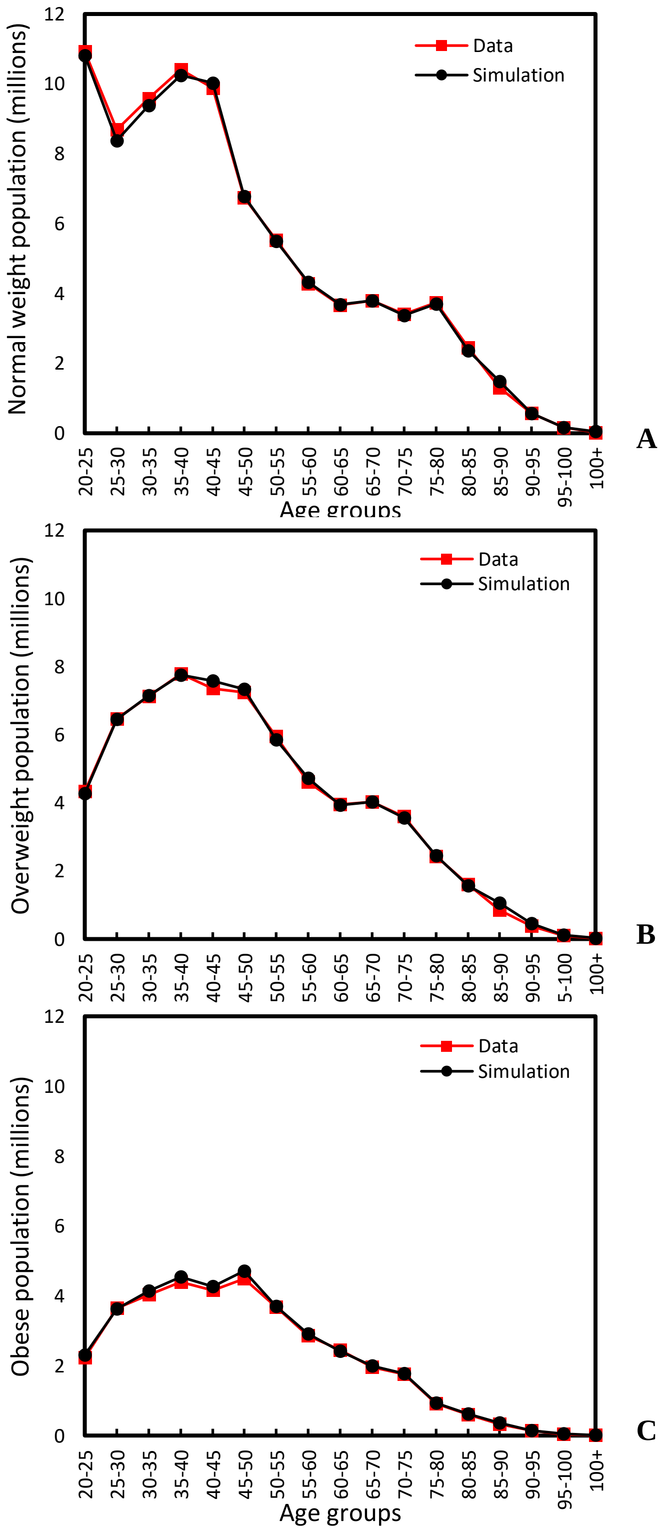

Supplement Figure S2. Comparing U.S. adult population in 1997 and the simulated population across age and by body mass index (BMI) status. A: Individuals with normal BMI (20-25 kg/m^2); B: Individuals with overweight BMI (25-30 kg/m^2); C: Individuals with obese BMI (≥30 kg/m^2)

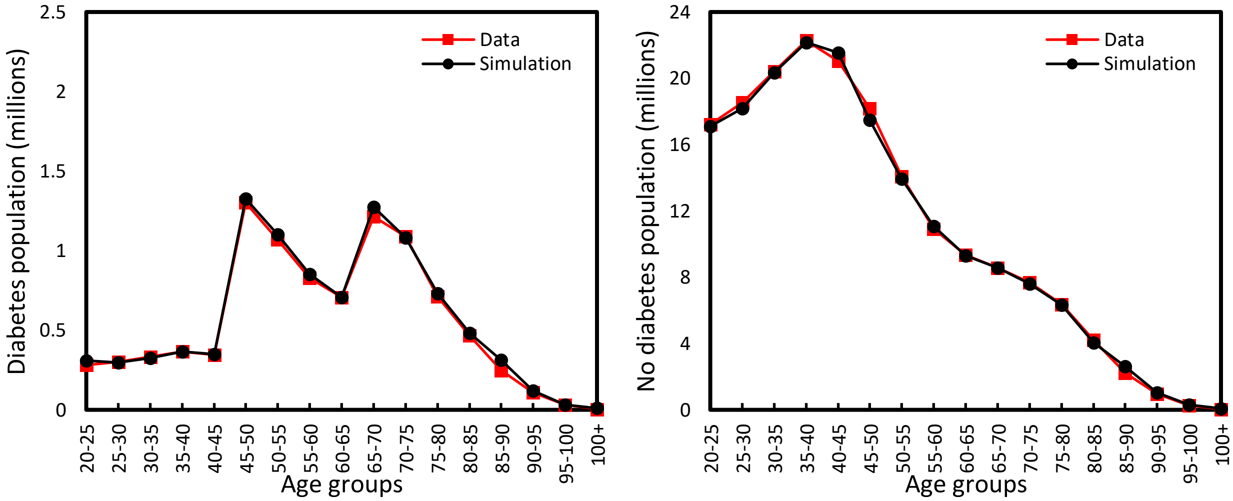

A **B**

Supplement Figure S3. Comparing U.S. adult population in 1997 and the simulated population across age and by Diabetes status. A: Individuals with diabetes; B: Individuals without diabetes

Validation of Birth Module

Supplement Table S2. Compare the simulated diabetes incidence to the reproted diabetes incidence in the U.S. population between 1997 and 2010

Year	Reported diabetes incidence (%)	Simulated diabetes incidence (%)	Difference (in %)
1997	0.48	0.45	9.07
1998	0.46	0.54	15.35
1999	0.49	0.56	14.79
2000	0.54	0.59	9.31
2001	0.59	0.60	1.33
2002	0.63	0.62	1.63
2003	0.64	0.65	1.36
2004	0.66	0.64	3.54
2005	0.68	0.70	2.98
2006	0.70	0.73	3.60
2007	0.73	0.68	6.57
2008	0.78	0.74	5.08
2009	0.77	0.80	3.78
2010	0.74	0.76	2.63

Note: The number of new cases of diabetes is obtained from[4]

Supplement Table S3. Compare the simulated diabetes prevalence to the reproted diabetes prevalence in the U.S. population between 1997 and 2010

Year	Reported diabetes prevalence (%)	Simulated diabetes prevalence (%)	Difference (in %)
1997	4.79	4.96	3.66
1998	5.29	5.13	2.96
1999	5.53	5.34	3.39
2000	5.90	5.55	5.95
2001	6.27	5.77	8.03
2002	6.54	6.01	8.17
2003	6.81	6.27	7.97
2004	7.17	6.55	8.54
2005	7.55	6.79	10.12
2006	7.84	7.03	10.35
2007	8.13	7.34	9.62
2008	8.54	7.57	11.41
2009	8.99	7.86	12.56
2010	9.20	8.19	11.20

Note: The number of diagnosed diabetes cases is obtained from[4]

Supplement Table S4. Compare the simulated prediabetes prevalence to the reproted prediabetes prevalence in the U.S. population between 2005 and 2010

Year	Prediabetes prevalence (%)	Simulated prediabetes prevalence (%)	Difference (%)
2005-2008	35.00	32.99	2.01
2005-2006	29.50	32.68	3.18
2010	33.00	33.97	0.97

Note: The diabetes prevalence data is obtained from[5–7]

Supplement Table S5. Compare the simulated death cases to the reproted death cases in the U.S. population between 1997 and 2010

Year	Total death cases (N)	Simulated total death cases (N)	Difference (%)
1997	2,314,245	2,476,400	7.006
1998	2,337,256	2,508,400	7.322
1999	2,391,399	2,573,150	7.600
2000	2,403,351	2,582,550	7.456
2001	2,416,425	2,571,650	6.423
2002	2,443,387	2,607,650	6.722
2003	2,448,288	2,629,200	7.389
2004	2,397,615	2,616,684	9.136
2005	2,448,017	2,773,736	13.31
2006	2,426,264	2,748,578	13.28
2007	2,423,712	2,773,947	14.45
2008	2,471,984	2,814,894	13.87
2009	2,437,163	2,780,315	14.08
2010	2,468,435	2,826,315	14.50

Note: The number of death cases is obtained from[4]

Mortality Module Validation

Supplement Table S6. Compare the simulated life expectancies to the reproted life expectancies for "No Diabetes" and "Prediabetes" individuals in the U.S. population between 1997 and 2010, by gender

Age group	Male expected age (year)	Simulated male expected age (year)	Difference (%)	Female expected age (year)	Simulated female expected age (year)	Difference (%)
20-25	52.86	52.88	0.04	58.26	58.89	1.08
25-30	48.24	48.45	0.44	53.42	53.71	0.55
30-35	43.58	43.81	0.53	48.58	48.72	0.30
35-40	38.98	39.07	0.25	43.78	44.19	0.95
40-45	34.42	34.74	0.95	39.06	39.31	0.64
45-50	30.04	30.52	1.59	34.44	35.00	1.63
50-55	25.76	26.17	1.60	29.92	30.36	1.48
55-60	21.72	22.17	2.11	25.58	25.95	1.45
60-65	18.00	18.31	1.72	21.50	22.08	2.71
65-70	14.58	15.20	4.30	17.68	18.31	3.60
70-75	11.54	12.12	5.07	14.12	15.04	6.54
75-80	8.90	9.20	3.42	10.90	11.81	8.40
80-85	6.62	7.08	6.98	8.06	8.95	11.13
85-90	4.88	5.50	12.70	5.80	6.74	16.20
90-95	3.60	4.05	12.37	4.12	5.03	22.16
95-100	3.22	1.89	41.15	2.98	3.16	6.07

Note: The mortality rates are generated according to the mortality CDF of U.S. adult population[8]

Supplement Table S7. Compare the simulated life expectancies to the reproted life expectancies for "Diabetes" individuals in the U.S. population between 1997 and 2010, by gender

Age group	Male expected age (year)	Simulated male expected age (year)	Difference (%)	Female expected age (year)	Simulated female expected age (year)	Difference (%)
20-25	40.41	40.01	0.96	35.70	35.65	0.11
25-30	35.52	35.66	0.46	31.00	31.87	2.83
30-35	32.14	32.06	0.12	29.11	28.19	3.10
35-40	27.34	28.82	5.60	24.51	24.98	1.97
40-45	24.82	24.99	0.78	22.87	21.71	4.76
45-50	20.17	20.18	0.41	18.45	18.80	2.19
50-55	17.86	17.45	1.95	16.68	16.23	2.19
55-60	13.56	13.80	2.26	12.53	12.66	1.34
60-65	12.55	12.22	1.91	10.74	10.93	2.22
65-70	8.28	8.48	3.51	7.36	7.63	4.52
70-75	7.65	7.44	1.98	6.29	6.73	8.67
75-80	4.47	5.16	17.42	4.10	4.89	19.48

Note: The mortality rates are generated according to a validated Cox proportional-hazard model[9]

Supplement B. Data Sets and Calculations

Birth Module

Birth rate is calculated by the following formula:

$$\text{Birth Rate} = \frac{(\text{Beginning Population} - \text{Previous Year End Population})}{(\text{Beginning Population})},$$

where beginning population and end population consist of only adult individuals. The adult population is calculated by intercensal estimates population associated with each year. These calculations are reported in the following table.

Supplement Table S8. “Birth” rate calculations

Year	Beginning population	Number of deaths	Final population	“Birth” rate (%)
1997	194,110,000	2,314,245	191,795,755	-----
1998	196,533,000	2,337,256	194,195,744	2.41
1999	199,000,000	2,391,399	196,608,601	2.41
2000	201,587,000	2,403,351	199,183,649	2.46
2001	204,062,000	2,416,425	201,645,575	2.39
2002	206,451,000	2,443,387	204,007,613	2.32
2003	208,682,000	2,448,288	206,233,712	2.24
2004	211,050,000	2,397,615	208,652,385	2.28
2005	213,511,000	2,448,017	211,062,983	2.27
2006	216,055,000	2,426,264	213,628,736	2.31
2007	218,481,000	2,423,712	216,057,288	2.22
2008	220,975,000	2,471,984	218,503,016	2.22
2009	223,491,000	2,437,163	221,053,837	2.23
2010	226,113,000	2,468,435	223,644,565	2.23

Note: Number of deaths is obtained from[4]

Age/BMI Module

The data for 1999-2002, 2003-2006, and 2007-2010 BMI information for male and female population are obtained from [1–3]. The BMI regression equation is then determined from the data as follows

-BMI regression equation for males in each year:

$$BMI_{new} = 27.76667 + 0.1\ \text{BMI}_{old}$$

-BMI regression equation for females in each year:

$$BMI_{new} = 28.025 + 0.1\ \text{BMI}_{old}$$

Supplement Table S9. Average BMI in U.S. population by gender

Years	Male	Female
1999-2002	27.8	28.1
2003-2006	28.4	28.4
2007-2010	28.6	28.7

Note: The data for 1999-2002, 2003-2006, and 2007-2010 BMI information for male and female population are obtained from[1–3].

Note that the expected value of the probability distribution is equal to average BMI change observed in the data. This module assumes that the BMI change depends only on gender. However, the BMI change over time depends on other risk factors and extending the module in this direction may impact the results.

Diabetes Progression Module

To mimic the natural progress of diabetes, we only considered data of individuals who participated in the control group of the PREMIER study (N=273)[10]. Participants in this group received information but no behavioral counseling on weight loss, increased physical activity, sodium reduction, and the DASH (Dietary Approaches to Stop Hypertension) dietary pattern. Estimating transition probability matrices is challenging because of irregular observation times, incomplete data points, and censored observations[11]. To overcome this difficulty, we first used a smoothing spline technique to fit a continuous curve to discrete observations over time to capture the variability in the data. Individuals with one observation are eliminated; the fasting blood glucose level progression for those with two observations are fitted by a least-squares linear model; and cubic smoothing splines[12] was used for individuals with three observations. Thereafter, we partitioned the curves into monthly intervals and estimated the fasting blood glucose level in each month. We then categorized each data point to “No Diabetes”, “Prediabetes”, and “Diabetes” states based on the fasting blood glucose level produced by the fitted curve and enumerated all pairs of

transitions across all curves, i.e., (levels at the start of a period, levels at the start of next period). Finally, we used a maximum likelihood estimator to calculate the TPM of movement between fasting blood glucose level categories[13]. Ideally, one expects to estimate TPMs for all combinations of age, gender, and BMI categories. However, because the number of individuals in the PREMIER data set is limited, estimating TPMs for each combination was impractical. Therefore, we settled to estimate a single TPM for fasting blood glucose level natural history model and calibrated it to determine a transitional probability matrix for each BMI class (normal weight, overweight, and obese). To that end, we used the risk ratio of being diagnosed with prediabetes and diabetes for overweight and obese individuals to adjust the corresponding TPMs[9] (Supplement B, Supplement Table S10).

Supplement Table S10. Cumulative probabilities for diabetes progression from one diabetes status to another were calculated according to the PREMIER trial[14]

	Diabetes Status	No diabetes	Prediabetes	Diabetes
Normal BMI (20-25 kg/m^2)	No diabetes	0.980	1.000	1.000
	Prediabetes	0.050	1.000	1.000
	Diabetes	0	0.015	1.000
Overweight BMI (25-30 kg/m^2)	No diabetes	0.965	1.000	1.000
	Prediabetes	0.010	0.982	1.000
	Diabetes	0	0.005	1.000
Obese BMI (≥kg/m^2)	No diabetes	0.945	1.000	1.000
	Prediabetes	0	0.960	1.000
	Diabetes	0	0.0025	1.000

Supplement C. Additional results

Lifestyle Interventions 15 Years Results

In reporting the results for **Table 1** and throughout the paper, all interventions are applied to the same sub-population. The inclusion criteria for this sub-population is an age between 25-65 years, BMI of at least 25, and an elevated level of fasting blood glucose level (prediabetes). However, the inclusion criteria for each intervention is different in the original study. In particular, the inclusion criteria for DPP intervention are an age of at least 25 years, a BMI of at least 24 and elevated fasting blood glucose level. In DPP-YMCA, the inclusion criteria are an age of at least 20 years, a BMI of at least 24 and elevated fasting blood glucose level. The inclusion criteria in the original study for HELP-PD intervention are a BMI of at least 25 and an elevated fasting blood glucose level. However, the original study does not specify an age limit. Thus, we considered the entire simulated population who are at least 20 years old. **Supplement Table S11** shows performance measures similar to those reported in **Table 1** for each intervention where the original inclusion criteria were considered. Note that the achievement rate discussed in the **Method** section have also been applied here.

Supplement Table S11. Differences in diabetes incidence, prevalence, and mortality rate of life-style interventions compared to no intervention in U.S. adult population after 5, 10, and 15 years using the study original criteria.

	Intervention type	5 years	10 years	15 years
Diabetes incidence (%)	DPP	0.07	0.03	0.03
	DPP-YMCA	0.04	0.02	0.02
	HELP-PD	0.07	0.04	0.04
Diabetes prevalence (%)	DPP	0.19	0.37	0.38
	DPP-YMCA	0.12	0.25	0.27
	HELP-PD	0.21	0.39	0.40
Diabetes mortality (%)	DPP	0.00	0.01	0.03
	DPP-YMCA	0.00	0.06	0.02
	HELP-PD	0.01	0.01	0.03

Long-Term (30 years) Life-style Intervention Results

Similar to **Table 2**, **Supplement Table S12** shows the long term (10 years) cost-effectiveness results of all three lifestyle interventions.

Supplement Table S12. 30 years impacts and cost-effectiveness analysis for life-style interventions among individuals with prediabetes

	Intervention type		
	DPP	DPP-YMCA	HELP-PD
Age group		***55-65 years***	
Diabetes averted (1000s)	305.14	196.23	325.20
Death averted (1000s)	290.32	141.98	313.20
Gain in diabetes-free survival time (Years)	0.05	0.03	0.05
Intervention cost ($1000)	31,074,700	7,677,100	10,039,300
Aversion savings ($1000)	25,997,928	16,718,796	27,707,040
Total savings ($1000)	-5,076,772	9,041,696	17,667,740
Age group		***45-65 years***	
Diabetes averted (1000s)	751.64	395.25	874.00
Death averted (1000s)	611.42	368.59	747.60
Gain in diabetes-free survival time (Years)	0.12	0.07	0.13
Intervention cost ($1000)	62,174,800	15,361,400	20,088,000
Aversion savings ($1000)	67,410,240	33,675,300	74,464,800
Total savings ($1000)	5,235,440	18,313,900	54,376,800
Age group		***35-65 years***	
Diabetes averted (1000s)	1200.04	634.26	1425.20
Death averted (1000s)	850.82	486.39	954.80
Gain in diabetes-free survival time (Years)	0.18	0.10	0.20
Intervention cost ($1000)	92,405,900	22,829,300	29,853,700
Aversion savings ($1000)	102,243,408	54,038,952	121,427,040
Total savings ($1000)	9,837,508	31,209,652	91,573,340
Age group		***25-65***	
Diabetes averted (1000s)	1627.92	852.81	1807.20
Death averted (1000s)	1069.32	573.19	1126.80
Gain in diabetes-free survival time (years)	0.24	0.14	0.26
Intervention cost ($1000)	126,929,900	31,358,600	41,007,400
Aversion savings ($1000)	138,698,784	72,659,412	153,973,440
Total savings ($1000)	11,768,884	41,300,812	112,966,040

References

1. McDowell, M. A. & National Center for Health Statistics. *Anthropometric reference data for children and adults: US population, 1999-2002*. (US Department of Health and Human Services, Centers for Disease Control and Prevention, National Center for Health Statistics, 2005).
2. McDowell, M. A. & National Center for Health Statistics. *Anthropometric reference data for children and adults: United States, 2003-2006*. (US Department of Health and Human Services, Centers for Disease Control and Prevention, National Center for Health Statistics, 2008).
3. Fryar, C. D., Gu, Q. & Ogden, C. L. Anthropometric reference data for children and adults: United States, 2007-2010. *Vital and Health Statistics. Series 11, Data from the national health survey* 1–48 (2012).
4. Murphy, S. L., Xu, J. & Kochanek, K. D. Deaths: final data for 2010. *National Vital Statistics Reports* **61**, 1–117 (2013).
5. Centers for Disease Control and Prevention. *National diabetes fact sheet: national estimates and general information on diabetes and prediabetes in the United States, 2011*. (US Department of Health and Human Services, Centers for Disease Control and Prevention, 2011).
6. Cowie, C. C. *et al.* Prevalence of diabetes and high risk for diabetes using A1C criteria in the US population in 1988–2006. *Diabetes Care* **33**, 562–568 (2010).
7. Centers for Disease Control and Prevention. *Diabetes report card 2012*. (US Department of Health Human Services, Centers for Disease Control and Prevention, 2012).
8. U.S. Census Bureau. Resident Population Estimates of the United States by Age and Sex: April 1, 1990 to July 1, 1999, with Short-Term Projection to November 1, 2000. (2001). Available at: https://www.census.gov/population/estimates/nation/intfile2-1.txt.
9. Yang, X. *et al.* Development and validation of an all-cause mortality risk score in type 2 diabetes: The Hong Kong Diabetes Registry. *Archives of Internal Medicine* **168**, 451–457 (2008).

10. Svetkey, L. P. *et al.* Premier: a clinical trial of comprehensive lifestyle modification for blood pressure control: rationale, design and baseline characteristics. *Annals of Epidemiology* **13**, 462–471 (2003).
11. Isaman, D. J., Herman, W. H. & Brown, M. B. A discrete-state discrete-time model using indirect observation. *Statistics in Medicine* **25**, 1035–1049 (2006).
12. Green, P. J. & Silverman, B. W. *Nonparametric regression and generalized linear models: a roughness penalty approach*. (CRC Press, 1993).
13. Ross, S. M. *Introduction to probability models*. (Academic Press, 2014).
14. Elmer, P. J. *et al.* Effects of comprehensive lifestyle modification on diet, weight, physical fitness, and blood pressure control: 18-month results of a randomized trial. *Annals of Internal Medicine* **144**, 485–495 (2006).
